# Impact of COVID Pandemic on Financial Burdens in Metro and Non-Metro Regions: Findings from a Cross-Sectional Survey

**DOI:** 10.21203/rs.3.rs-6874039/v1

**Published:** 2025-07-15

**Authors:** Jingbo Yi, Mohamed I Elsaid, Cecelia DeGraffinreid, Electra D. Paskett

**Affiliations:** The Ohio State University Comprehensive Cancer Center; The Ohio State University; The Ohio State University Comprehensive Cancer Center; The Ohio State University Comprehensive Cancer Center

**Keywords:** Rural, metro, COVID-19, financial burden, unemployment

## Abstract

**Purpose:**

The impact of the COVID-19 pandemic on employment and various financial burden domains has not been well researched, particularly in vulnerable populations. This study examined the impact of the pandemic among those living in an Ohio metro county compared to a non-metro county on employment and financial concerns.

**Methods:**

We conducted a survey between June and November 2020 and collected participants’ demographics, employment status, perceived financial status, and health-related conditions. Multivariable logistic regression models assessed the association between regions of residence (metro or non-metro) and various perceived financial concerns.

**Findings:**

Among the 6,123 respondents with complete data, the average age was 55.7 years, 67.2% were female, 87.1% were white non-Hispanic, and 30.2% lived in a non-metro county. Compared to those living in metro counties, non-metro respondents had significantly higher odds of reporting not having full- or part-time employment right before the start of the lockdown (aOR=1.24; 95% Cl 1.02–1.52) and significantly higher odds of being unpaid for a full- or part-time job (aOR=1.5; 95%C11.23–1.84) during the pandemic. Region of residence did not significantly differ in terms of employment loss or concern regarding financial outcomes.

**Conclusions:**

Although metro and non-metro residents did not differ in employment loss due to the COVID-19 pandemic, non-metro residents had a higher likelihood of not having a full- or part-time job prior to the pandemic and remained more likely to be unpaid during the pandemic. However, nonmetro residents reported fewer financial concerns, although statistically insignificant, highlighting potential differences in perception and cognitive processes.

## INTRODUCTION

The onset of the COVID-19 pandemic has led to complex economic and financial changes for residents of the United States. Many secondary impacts of COVID-19 are associated with the social determinants of health, including poverty, built environment, race, and rurality ^1–4^ The Census Bureau’s Household Pulse Survey estimated that close to half of all households experienced loss of employment in the early months of the pandemic, disproportionately affecting households with children and low-income households^5^. The consequences of employment and financial loss can be severe, including changes in long-term financial and retirement plans, missed economic and employment opportunities, and prolonged difficulty in paying bills and meeting basic needs ^6–8^.

Job loss from the pandemic was associated with large metropolitan areas, which persisted even after COVID-19 infection rates rose in less populous areas ^9^. In contrast, rural communities were more susceptible to negative health effects of the pandemic. Rural communities are characterized by older, less educated, and lower socioeconomic class residents with limited access to healthcare and poorer health behaviors than urban communities ^10–12^. However, research on the burden of COVID-19 mortality by rurality is limited. Although some studies show an elevated risk of COVID-19 mortality in either urban or rural areas, other studies suggest more granularity in terms of geographical representation, as some non-metro residences present a similar risk as major metropolitan areas ^1,13,14^. Regardless of the heterogeneous COVID-19 mortality rates, residents of rural areas remain vulnerable to the secondary impacts of COVID-19, given their relatively older age, lower socioeconomic class, less access to healthcare facilities, and greater reliance on social safety nets ^15–17^.

Despite the increased literature characterizing pandemic-related financial loss and notable differences in health risks between rural and urban areas, very few studies have compared different domains of employment and financial loss between the two communities. This study examines the differences in the impact of COVID-19 pandemic on employment and financial concerns between metro and non-metro residents.

## METHODS

### Overview

This study was part of an NCI-funded initiative conducted in conjunction with 16 other NCI-designated Cancer Centers, the IC-4 (Impact of COVID-19 on the Cancer Continuum Consortium). The initiative was funded to work collectively to develop core survey items and implement population surveys in the respective catchment areas. The overall goal of the IC-4 was to assess how differences in demographics (rural/urban, age, gender, race, educational attainment) impact engagement in cancer preventive behaviors (e.g., tobacco cessation, screening, diet) and cancer management/survivorship behaviors (e.g., adherence to treatment, adherence to surveillance, access to health services) in the context of COVID-19 environmental constraints (e.g., social distancing, employment, mental health). Each site had its own theoretical framework and survey method. Our site used the IC-4 core set of common data elements augmented with issues specific to Ohio, with remote data collection methods to include many unique and diverse populations. This study was approved by the OSU Institutional Review Board (June 2020).

### Theoretical Framework:

This study was grounded in the Health Belief Model (HBM)^18,19^. According to the HBM, individual changes in health behaviors depend on a series of health beliefs, including : 1) perceived susceptibility to COVID-19 exposure, 2) perceived severity of the consequences of contracting COVID-19 (e.g., hospitalization or death), 3) perceived benefits of the effectiveness of the proposed COVID-19 prevention measures, 4) perceived barriers to executing the proposed prevention measures, 5) cues to the proposed prevention actions, and 6) self-efficacy in the person’s ability to successfully perform COVID-19 prevention measures.

### Setting

The study was conducted in The Ohio State University Comprehensive Cancer Center (OSUCCC) catchment area. Participants who agreed to be re-contacted from previous studies were asked to participate in this study, with a wide variety of populations, including healthy residents and cancer patients. Ohio was one of the first states to implement a statewide stay-at-home order. In addition, Ohio has many diverse populations, including 50 of the 88 counties that are designated as rural, and 32 counties are part of the Appalachian area of the US. Both areas are healthcare professional shortage areas with poor access to the internet and cell phone coverage, limiting the availability of good and accurate information about COVID-19. Ohio’s urban areas have higher proportions of minorities, both established and new immigrants, many of whom do not speak English and have relied on the sociality of their community for support and information in the past.

### Survey Development

The survey elements (S1 table) were finalized in conjunction with other members of the IC-4 ^20^. The survey included individual behaviors related to the mitigation of COVID-19 transmission, the challenges related to social distancing, self/family isolation, stress, and health behaviors that are highly relevant to cancer and other chronic diseases (i.e., type, duration, and location of physical activity, tobacco/marijuana or alcohol use, vaping/e-cig use, exposure to secondhand smoke, nutrition/diet, health information-seeking and participation in clinical trials, and access to health services). Questions also assessed perceived stigma related to COVID-19 with respect to different population groups and covariates, such as health and mental health, which were suspected to moderate these influences. Moreover, we assessed the impact of children being out of school and employment challenges (i.e., remote working and unemployment) as well as the influence of social media on information, knowledge, behaviors, and attitudes.

### Sample Selection

Eligible participants were adults aged ≥ 18 years who consented to participate in the study. To ensure the inclusion of the most vulnerable, underserved, and minority populations, we sought to recruit healthy adult volunteers, cancer patients, cancer survivors, and cancer patients and survivors’ caregivers in our catchment area in Ohio and Indiana. This was achieved by employing two recruitment strategies. First, we identified and contacted individuals who had previously participated in studies conducted at OSU and consented to be contacted for future research projects. In addition, we invited cancer patients and survivors who completed the study survey to nominate their primary caregivers to participate in this study. The list of previous research projects conducted at OSU included the Rural Interventions for Screening Effectiveness (RISE) study (R01 CA196243), the Community Initiative Towards Improving Equity and Health Status (CITIES) cohort (Supplement to P30CA016058), the Buckeye Teen Health Study (BTHS) study (P50CA180908), the Ohio State University Center of Excellence in Regulatory Tobacco Science (CERTS) cohort (P50CA180908), and members of the Total Cancer Care (TCC) cohort (P30CA016058). Second, to further enhance the representativeness of our study sample and ensure the inclusion of minority and underserved communities, we utilized our community partners and the OSUCCC Pelotonia listservs to recruit participants.

### Interview/Data Collection

We utilized several data collection methods, including web, phone, and mail surveys. Respondents with valid email addresses received an initial survey invitation email along with three reminders seven days apart. All participants were initially screened using an eligibility form to confirm their current residence in Ohio or Indiana before the survey was conducted. Participants were able to save the web survey and resume it later. Those who partially completed the web survey received an email reminder one week after they last accessed it. A trained interviewer contacted participants without an email address and those with invalid emails via phone. Participants who were initially contacted by phone were offered the option to complete the survey over the phone or online. We mailed a cover letter and paper survey with a self-addressed, stamped return envelope to participants who requested a mailed survey. For non-English-speaking participants, a bilingual staff member administered the survey in the appropriate language. Participants were offered a $10 gift card upon completion of the survey. All data were collected and managed using the Research Electronic Data Capture (REDCap) secure web-based application hosted at OSU from June 19, 2020, to November 30, 2020.

### Study Measures

**Figure 1:**
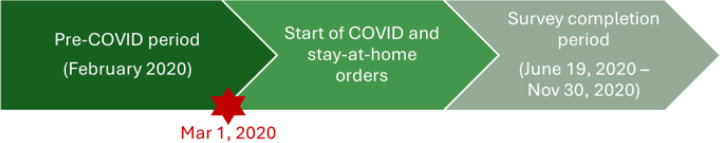
COVID events and study timeline

The exposure of interest in this study was the region of residence of the respondents. We used the respondents’ zip codes to classify them as either metro or nonmetro residences based on the 2013 Rural Urban Continuum Codes (RUCC 2013). The primary outcomes were three employment characteristics related to COVID-19: occupational status pre-COVID-19, job loss due to COVID-19, and payment for a job during the pandemic. Figure 1 summarizes the timeline of COVID events and study completion. For occupation status pre-COVID-19, participants were asked “*Which category best describes your occupational status in February 2020 prior to the stay-at-home orders put in place as a result of the COVID-19 pandemic?”* Responses were grouped as employed full- or part-time, retired, and non-employment categories, including unemployed, homemaker, student, disabled, and all others. For job loss due to COVID-19, participants were classified as “Yes” and “No” based on their responses to the question: *Since March 1st, did you lose a job because of COVID-19?* For payment status for a job during the pandemic, respondents were classified as currently unpaid if they indicated “No” to the question: *Are you currently being paid for a full- or part-time job, including being paid by a job while you stay home?*

The secondary outcome of interest was perceived financial and livelihood burdens. Responses to the following five domains (0–3 points each) regarding participants’ perception of the past 30 days prior to survey completion were recorded to obtain an aggregate score (0–15): personal financial loss, making rent or mortgage payments, providing for oneself or family, having enough food for oneself or family, and not having enough basic supplies. The aggregate score was also assessed as a categorical outcome based on the tertiles, where low financial concern was defined as a score of 0, moderate concern as a score of 0–2, and high concern as a score of 3 or higher.

### Statistical Analysis

Descriptive statistics were used to summarize the respondent characteristics, including means and standard deviations (SD) for continuous variables and frequency and proportions for categorical variables. Differences in characteristics between metro and non-metro participants and bivariate analyses of the outcomes were assessed using the Kruskal-Wallis and chi-square tests for continuous and categorical variables, respectively. We used multivariable binary and multinomial logistic regression models to assess the associations between the region of residence and each of the employment and financial burden outcomes. The models were adjusted for several baseline covariates, including age, sex, race/ethnicity, marital status, health insurance, education, state, household income, having prior cancer, number of adults and children in the household, primary caregiver status, and health status. Complete case analyses were performed in this study. All statistical analyses were conducted using SAS version 9.4, with two-tailed tests and a significance level of 0.05.

## RESULTS

**Figure 2:**
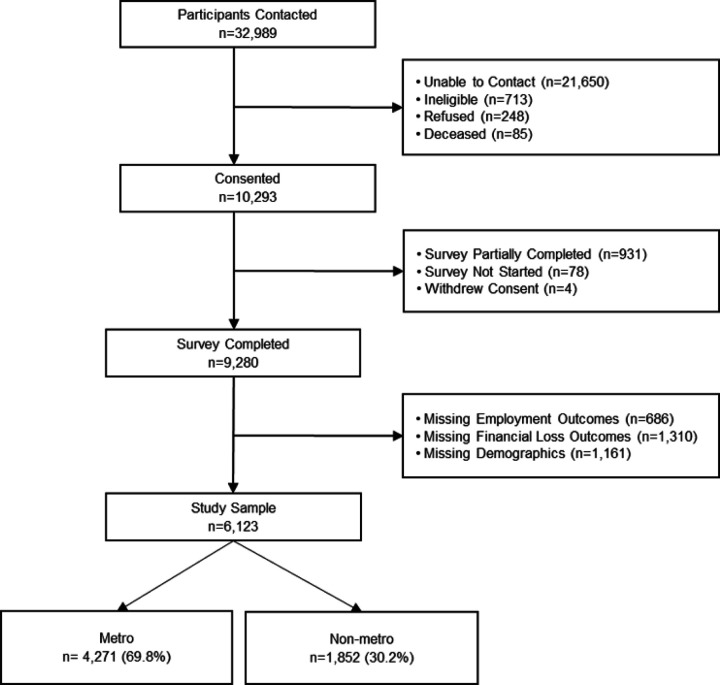
Study Schema

The analytical sample included 6,123 respondents (Fig. 2). As described in Table 1, most respondents lived in a metropolitan region (69.8%), and the median age was 57 years. The majority of respondents were female (67.2%), white non-Hispanic (87.1%), college-educated (57.4%), and married (74%). Respondent characteristics significantly differed for metro and non-metro residents, except for caregiving status and self-reported health status. Non-metro residence was associated with older age, female sex, being white non-Hispanic, less education, public or no insurance, less household income, more adults and less children in the household, and no history of cancer.

In the unadjusted analyses, compared to metropolitan residents, non-metro residents were significantly less likely to be employed prior to the start of the COVID-19 lockdowns (Table 2). Among respondents who were employed full or part time pre-COVID-19, non-metro residents were more likely to be unpaid during the pandemic at the time of the survey (32.2% vs. 22.2%, p < 0.0001). However, job loss due to COVID-19 was not associated with the region of residence. In addition, none of the concerns regarding financial or livelihood outcomes were significantly associated with the region of residence in the bivariate analyses.

**Figure 3:**
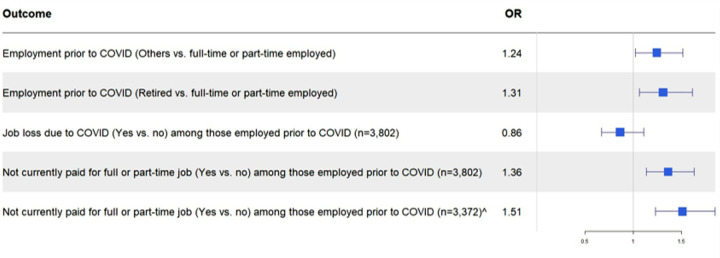
Multivariable models for employment outcomes OR, Odds ratio; Odds ratios for employment outcomes comparing non-metro to metro (reference) residents; Logistic regression models adjusted for: age, sex, race-ethnicity, marital status, health insurance, education, state, household income, having prior cancer, number of adults and children in household, primary caregiver status, and health status. Other employment status includes those who are unemployed, homemakers, students, disabled, and all other categories. ^˄^Among participants who additionally did not report job loss due to COVID.

Figure 3 summarizes the adjusted odds ratios (aOR) for each employment outcome. Compared with metro residents, non-metro residents had higher odds of not being full- or part-time employed [aOR for being retired, 1.31 (1.06–1.61); aOR for other non-employed categories, 1.24 (1.02–1.52)]. The adjusted odds of job loss due to COVID were not significantly different between regions of residence. However, among those who were employed prior to COVID, non-metro residents also had higher odds of not currently being paid for any job at the time of the survey during the pandemic [aOR (95%Cl): 1.36 (1.14–1.63)]. This trend was consistent in subgroup analyses of respondents who did not report job loss due to COVID-19 [aOR (95%Cl), 1.51 (1.23–1.85)]. In sensitivity analyses where unemployment was assessed as a separate outcome and in subgroup analyses, only those of working age (< 65 years old) were included, residence was no longer significantly associated with employment status prior to the pandemic (S2 Table). However, non-metro residents remained with significantly higher odds of being unpaid for a job during the early pandemic, among a subgroup of working-age residents [aOR (95%Cl): 1.39 (1.18–1.62)].

**Figure 4:**
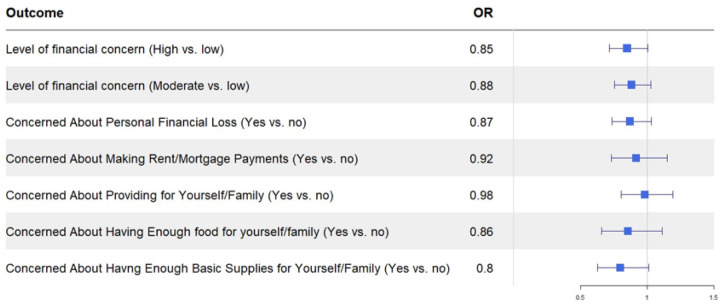
Multivariable models for financial burden outcomes OR, Odds ratio; Odds ratios for employment outcomes comparing non-metro to metro (reference) residents; Logistic regression models adjusted for: age, sex, race-ethnicity, marital status, health insurance, education, state, household income, having prior cancer, number of adults and children in household, primary caregiver status, and health status; Level of financial concern defined as “Low” for Financial Concern Score of 0, “Moderate” for Financial Concerns Scores of 1–2, or “High” for Financial Concern Scores of 3+.

In multivariable analyses, region of residence was not significantly associated with any of the financial burden outcomes, but the direction of effect showed that non-metro residents were less concerned about each outcome than metro residents. The greatest effect size was observed for concern about having enough basic supplies and food (aOR 0.80 and 0.86, respectively) (Fig. 4). Nonmetro residents had lower odds for having high level of financial concern compared to metro residence (aOR = 0.85), although the confidence interval was borderline insignificant (Cl: 0.72–1.01). In subsequent exploratory analyses of factors associated with continuous financial burden scores, region of residence remained borderline insignificant; however, younger age, non-white ethnicity, lower education and income, having public or no insurance, not having prior cancer, being a primary caregiver, and having poor self-perceived health were associated with higher financial concern scores (S3 Table).

## DISCUSSION

This study found that in a sample of Ohio and Indiana residents, non-metro respondents were more likely to not have a full- or part-time job prior to the pandemic and remained more likely to be unpaid fora full- or part-time job during the pandemic despite having similar rates of job loss due to the pandemic. In contrast, we also found a slightly protective effect of being a non-metro resident on the level of concern regarding various financial burdens. These findings highlight the complexity of rural health and the systemic factors that contribute to the diverse impact of the COVID-19 pandemic.

Our finding that non-metro residents have a higher likelihood of not being employed in a full- or part-time job right before the pandemic is consistent with nationwide data^21^. Federal records showed that during the 5-year period right before and during the pandemic, employment rates were significantly higher across all age groups and genders in metro counties, and non-metro communities did not hit the same employment rate before the Great Recession when the COVID-19 pandemic first hit. However, this overall trend may obscure several differential trends by industry, region, and poverty level. Our observed statistical effect was no longer significant in the sensitivity analyses when unemployment was separated from all the other non-full- or part-time employment categories. This decrease in statistical significance may reflect the volatility of the differences between self-reported unemployment and other non-employment categories, including homemakers, students, and disability. As the National Bureau of Economic Research finds, unemployment and employment loss rates vary greatly by industry and individual characteristics^22^. Women with young children are more likely to be absent from work but not more likely to be unemployed; single parents, who are disproportionately women, are more likely to experience job loss^22^. These differences in terminology may contribute to the difference between the observed effect of non-metro residence on any non-employment categories compared to the effect on unemployment separated from specific categories (such as homemakers).

In this study, the impact of the pandemic on employment outcomes was multifaceted. Among respondents who had a full- or part-time job before the pandemic, non-metro residents were less likely to experience job loss due to COVID, although the difference was not statistically significant. This protective effect has been frequently reported in the literature and is partly attributable to the policy changes enforced in population-dense areas^9,23,24^. However, our analyses showed that non-metro residents were also significantly more likely to be unpaid during the pandemic. We theorize that this contrast is attributable to income loss that participants did not perceive to be directly related to job loss from COVID. For example, Greteman et al. (2022) found that compared to urban respondents, rural respondents reported more frequently finding it difficult to get by on their household’s income during the pandemic, but also reported less frequency of income loss *due* to COVID-19^24^. From the Household Pulse Survey data on employment, Anyamele (2021) found that employment income loss was greater for those who could telework compared to those who could not, and telework options were more prevalent in metro areas^25^. Thus, further evidence is needed to explore the heterogeneity of the non-metro population and the factors contributing to income loss compared to employment loss.

Non-metro residents also reported less concern regarding each of the financial and wellbeing outcomes, including personal financial loss, paying rent/mortgage, providing for self/family, and having enough food or supplies for self/family. One potential explanation is that non-metro counties were less likely to participate or engage in COVID-related policies and therefore less likely to experience emotional distress from COVID-related policies. Studies have found that rural communities across the US were less likely to participate in social distancing and mask wearing, issue stay-at-home orders, and have school, store, and industry closures^15,26–29^. As a previous survey in Iowa also found, rural residents were less likely to perceive pandemic-related disruptions to their daily lives and less likely to report negative emotional reactions^24^. These factors in rural residences may be protective against psychological stress of the pandemic, at least in the initial few months to one year of the stay-at-home orders.

### Limitations/strengths

This study had several strengths. First, the survey collected important data on sociodemographic factors known to be associated with the social determinants of health, particularly in COVID-19 disparities. Second, to our knowledge, this study is the first to assess urban-rural differences related to the COVID-19 financial impact in Ohio and Indiana, with a large proportion from the Appalachia population. Third, this study assessed a range of less-researched secondary effects of COVID-19, including employment and financial outcomes, and advances the evidence on secondary effects of COVID-19 in the literature.

The first limitation of our study is that our survey included self-reported elements, which introduced potential biases. Self-reported responses are susceptible to recall and social desirability bias, especially regarding financial well-being. In addition, selection bias potentially arises because our survey was not a random sample of Ohio/Indiana residents and not representative of the states, and the analyses included only participants who had complete responses to all exposure and outcome variables. Because data collection occurred during the first six months of the COVID surge, responses only represent outcomes in the early days of the pandemic. However, understanding responses early in the pandemic provides insights into its relative impact on overall trends and serves as a framework for better understanding early responses to pandemics. Finally, we note that rural areas are not homogeneous across geospatial levels, and interventions require multi-level considerations. Certain rural communities are more vulnerable to economic shocks and health crises based on community characteristics. For example, one geospatial analysis found that beyond the simple urban-rural classification, other socioeconomic qualities, such as population density, elderly residents, or poverty level, may better predict COVID-19 related outcomes^13^.

### Implications

Our study results underscore pre-existing economic inequities and highlight differences in financial perception and cognitive processes between people living in metropolitan and non-metropolitan areas of residence in Ohio. These contrasting findings further suggest the importance of multilevel intervention strategies to address different domains of inequality. These disparities prompt further research on the long-term financial and social impacts of COVID-19 on rural populations.

## Supplementary Material

Supplement 1

## Figures and Tables

**Table 1:** Characteristics of survey respondents by region of residence

Demographics	Nonmetro (N = 1,852)	Metro (N = 4,271)	Total (N = 6,123)	P-value
**Age, years**				0.0016^1^
Mean (SD)	56.6 (12.06)	55.3 (13.81)	55.7 (13.32)	
Median	57	56	57	
**Age, years,** n (%)				<.0001^2^
<35	112 (6.0%)	386 (9.0%)	498 (8.1%)	
35–49	304 (16.4%)	1055 (24.7%)	1359 (22.2%)	
50–59	630 (34.0%)	1025 (24.0%)	1655 (27.0%)	
60–69	580 (31.3%)	1102 (25.8%)	1682 (27.5%)	
70+	226 (12.3%)	703 (16.5%)	929 (15.2%)	
**Sex**, n (%)				<.0001^2^
Male	422 (22.8%)	1588 (37.2%)	2010 (32.8%)	
Female	1430 (77.2%)	2683 (62.8%)	4113 (67.2%)	
**Race and Ethnicity**, n (%)				<.0001^2^
White non-Hispanic	1734 (93.6%)	3601 (84.3%)	5335 (87.1%)	
Black non-Hispanic	34 (1.8%)	340 (8.0%)	374 (6.1%)	
Hispanic	27 (1.5%)	99 (2.3%)	126 (2.1%)	
Other non-Hispanic	57 (3.1%)	231 (5.4%)	288 (4.7%)	
**State**, n (%)				<.0001^2^
Indiana	200 (10.8%)	6 (0.1%)	206 (3.4%)	
Ohio	1652 (89.2%)	4265 (99.9%)	5917 (96.6%)	
**Education**, n (%)				<.0001^2^
Highschool or less	330 (17.8%)	422 (9.9%)	752 (12.3%)	
Some college/Associate’s degree	700 (37.8%)	1159 (27.1%)	1859 (30.4%)	
Bachelor’s degree	442 (23.9%)	1393 (32.6%)	1835 (30.0%)	
Master’s degree or higher	380 (20.5%)	1297 (30.4%)	1677 (27.4%)	
**Marital status**, n (%)				<.0001^2^
Single, never been married	118 (6.4%)	447 (10.5%)	565 (9.2%)	
Married/living as married	1407 (76.0%)	3123 (73.1%)	4530 (74.0%)	
Widowed, separated, divorced, or other	327 (17.7%)	701 (16.4%)	1028 (16.8%)	
**Health Insurance,** n (%)				<.0001^2^
No insurance	97 (5.2%)	114 (2.7%)	211 (3.4%)	
Public only	278 (15.0%)	592 (13.9%)	870 (14.2%)	
Private only	1050 (56.7%)	2520 (59.0%)	3570 (58.3%)	
Public and Private	427 (23.1%)	1045 (24.5%)	1472 (24.0%)	
**Household Income,** n (%)				**<**.0001^2^
<$35K	291 (15.7%)	523 (12.2%)	814 (13.3%)	
$35K-$49,999	228 (12.3%)	410 (9.6%)	638 (10.4%)	
$50K-$74,999	406 (21.9%)	691 (16.2%)	1097 (17.9%)	
$75K+	927 (50.1%)	2647 (62.0%)	3574 (58.4%)	
Caregiver^*^, n (%)				0.8430^2^
No	1699 (91.7%)	3909 (91.5%)	5608 (91.6%)	
Yes	147 (7.9%)	344 (8.1%)	491 (8.0%)	
**Number of adults in home**				0.0069^1^
Mean (SD)	2.2 (1.31)	2.1 (1.15)	2.1 (1.20)	
Median	2	2	2	
**Number of Children in home**				<.0001^1^
Mean (SD)	0.4 (0.94)	0.5 (0.93)	0.5 (0.93)	
Median	0	0	0	
**History of cancer,** n (%)				**<**.0001^2^
No	1162 (62.7%)	1969 (46.1%)	3131 (51.1%)	
Yes	690 (37.3%)	2302 (53.9%)	2992 (48.9%)	
**Self Reported Health Status,** n (%)				0.3286^2^
Excellent	209 (11.3%)	440 (10.3%)	649 (10.6%)	
Very good	647 (34.9%)	1421 (33.3%)	2068 (33.8%)	
Good	625 (33.7%)	1541 (36.1%)	2166 (35.4%)	
Fair	297 (16.0%)	683 (16.0%)	980 (16.0%)	
Poor	74 (4.0%)	186 (4.4%)	260 (4.2%)	

1 Kruskal-Wallis p-value

2 Chi-Square p-value;

* including 24 (0.6%) respondents who reported unknown.

**Table 2:** Employment and financial burden outcomes by region of residence

	Region of Residence		Total	P-value
	Nonmetro (N = 1852)	Metro (N = 4271)	(N = 6123)	
**Employment Characteristics**				
**Occupation Status Pre COVID-19,** n (%)				0.0003^1^
Employed full- part-time	1092 (59.0%)	2710 (63.5%)	3802 (62.1%)	
Retired	479 (25.9%)	1055 (24.7%)	1534 (25.1%)	
Other^¶^	281 (15.2%)	506 (11.8%)	787 (12.9%)	
Lost Job Due to COVID-19^*^, n (%)				0.6923^1^
Yes	127 (11.6%)	303 (11.2%)	430 (11.3%)	
Currently Unpaid^*^, n (%)				<.0001^1^
Yes	352 (32.2%)	602 (22.2%)	954 (25.1%)	
**Financial Characteristics**				
**Financial Concerns Score**†, n (%)				0.8777^1^
N	1852	4271	6123	
Mean (SD)	2.7 (3.17)	2.7 (3.23)	2.7 (3.21)	
Median (IQR)	2 (0–4)	2 (1–4)	2 (1–4)	
**Level of Financial Concern**††, n (%)				0.1605^1^
Low Concern	482 (26.0%)	1048 (24.5%)	1530 (25.0%)	
Moderate Concern	686 (37.0%)	1690 (39.6%)	2376 (38.8%)	
High Concern	684 (36.9%)	1533 (35.9%)	2217 (36.2%)	
**Worried About Personal Financial Loss,** n (%)				0.4299^1^
None or some of the time	1546 (83.5%)	3530 (82.7%)	5076 (82.9%)	
Most or all of the time	306 (16.5%)	741 (17.3%)	1047 (17.1%)	
**Worried About Making Rent or Mortgage Payment,** n (%)				0.5034^1^
None or some of the time	1680 (90.7%)	3897 (91.2%)	5577 (91.1%)	
Most or all of the time	172 (9.3%)	374 (8.8%)	546 (8.9%)	
**Worried About Providing for Yourself or Your Family,** n (%)				0.3886^1^
None or some of the time	1619 (87.4%)	3767 (88.2%)	5386 (88.0%)	
Most or all of the time	233 (12.6%)	504 (11.8%)	737 (12.0%)	
**Concerned About Having Enough Food for Yourself or Your Family,** n (%)				0.8320^1^
None or some of the time	1735 (93.7%)	3995 (93.5%)	5730 (93.6%)	
Most or all of the time	117 (6.3%)	276 (6.5%)	393 (6.4%)	
**Worried About Not Having Enough Basic Supplies,** n (%)				0.2952^1^
None or some of the time	1714 (92.5%)	3919 (91.8%)	5633 (92.0%)	
Most or all of the time	138 (7.5%)	352 (8.2%)	490 (8.0%)	

1 Chi-Square p-value;

* Among participants whose occupational status in February 2020 prior to the stay-at-home orders put in place were full-time or part-time employed (n = 3,802), non-metro (n = 1,092), metro (n = 2,710).

¶ Other includes those who are unemployed, homemakers, students, disabled, and all other categories

† Total financial concern score was an aggregate of response scores (0–15) to each of the following 5 concern domains (0–3):

personal financial loss, making rent or mortgage payment, providing for oneself or family, having enough food for oneself or family, and not having enough basic supplies;

†† Defined as “Low” for Financial Concern Score of 0, “Moderate” for Financial Concerns Scores of 1–2, or “High” for Financial Concern Scores of 3+

## Data Availability

Requests from researchers will be reviewed by the project Multiple Principal Investigators (MPIs), and we will provide the requesting researcher with the minimum necessary data; the shared data will not have any individual participant identifiers or specific clinic identifiers. The requesting researcher(s) will only use the data for the purposes for which it was requested and only by the individuals listed in the request. The data will be sent in a computer data file, and the requesting researcher(s) will be responsible for notifying the MPIs upon completion of analysis and indicate the manner in which the data were destroyed. Any presentations, abstracts, or publications created must include an acknowledgement/reference to the project and project team.

## References

[1] Paul R, Arif A, Pokhrel K, Ghosh S. The Association of Social Determinants of Health With COVID-19 Mortality in Rural and Urban Counties. J Rural Health. Mar 2021,37(2):278–286. doi:10.1111/jrh.12557 33619746 PMC8014225

[2] Tsai J, Wilson M. COVID-19: a potential public health problem for homeless populations. Lancet Public Health. Apr 2020;5(4):e186–e187. doi:10.1016/s2468-2667(20)30053-0 32171054 PMC7104053

[3] Yancy CW. COVID-19 and African Americans. JAMA. May 19 2020;323(19):1891–1892. doi:10.1001/jama.2020.6548 32293639

[4] Rollston R, Galea S. COVID-19 and the Social Determinants of Health. Am J Health Promot. Jul 2020;34(6):687–689. doi:10.1177/0890117120930536b 32551932

[5] COVID-19 Pandemic’s Impact on Household Employment and Income (Library of Congress) (2020).

[6] Reece S, Dickerson J, Kelly B, McEachan RRC, Pickett KE. The long-term impact of the Covid-19 pandemic on financial insecurity in vulnerable families: Findings from the Born in Bradford Covid-19 longitudinal study. PLoS One. 2023;18(11):e0295064. doi:10.1371/journal.pone.0295064 38019781 PMC10686492

[7] Parker K, Minkin R, Bennett J. Economic Fallout From COVID-19 Continues To Hit Lower-Income Americans the Hardest. 2020. https://www.pewresearch.org/social-trends/2020/09/24/economic-fallout-from-covid-19-continues-to-hit-lower-income-americans-the-hardest/#:~:text=Lower%2Dincome%20adults%20continue%20to,%25%20of%20upper%2Dincome%20adults.

[8] Bruce C, Gearing ME, DeMatteis J, Financial vulnerability and the impact of COVID-19 on American households. PLoS One. 2022;17(1):e0262301. doi:10.1371/journal.pone.0262301 35030175 PMC8759691

[9] Cho SJ, Lee JY, Winters JV. Employment impacts of the COVID-19 pandemic across metropolitan status and size. Growth Change. Dec 2021;52(4):1958–1996. doi:10.1111/grow.12540 34548677 PMC8444738

[10] Malatzky C, Cosgrave C, Gillespie J. The utility of conceptualisations of place and belonging in workforce retention: A proposal for future rural health research. Health Place. Mar 2020;62:102279. doi:10.1016/j.healthplace.2019.102279 32479357

[11] Hartley D. Rural health disparities, population health, and rural culture. Am J Public Health. Oct 2004;94(10):1675–8. doi:10.2105/ajph.94.10.1675 15451729 PMC1448513

[12] Taylor MM. Rural Health Disparities Public Health, Policy, and Planning Approaches. 1 ed. SpringerBriefs in Public Health. Springer Cham; 2019.

[13] Zhang CH, Schwartz GG. Spatial Disparities in Coronavirus Incidence and Mortality in the United States: An Ecological Analysis as of May 2020. J Rural Health. Jun 2020;36(3):433–445. doi:10.1111/jrh.12476 32543763 PMC7323165

[14] Grome HN, Raman R, Katz BD, Disparities in COVID-19 Mortality Rates: Implications for Rural Health Policy and Preparedness. J Public Health Manag Pract. Sep-Oct 01 2022;28(5):478–485. doi:10.1097/phh.0000000000001507 35389953 PMC9307261

[15] Monnat SM. Rural-Urban Variation in COVID-19 Experiences and Impacts among U.S. Working-Age Adults. Ann Am Acad Pol Soc Sci. Nov 2021;698(1):111–136. doi:10.1177/00027162211069717 35493266 PMC9055492

[16] Ranscombe P. Rural areas at risk during COVID-19 pandemic. Lancet Infect Dis. May 2020;20(5):545. doi:10.1016/s1473-3099(20)30301-7 PMC7164870 32311327

[17] Peng Y, Peng X, Yin M, He J, Ma L. The welfare effects of impoverished rural areas: Review and research prospects. Heliyon. Sep 2023;9(9):e19513. doi:10.1016/j.heliyon.2023.e19513 37809881 PMC10558749

[18] Janz NK, Becker MH. The Health Belief Model: a decade later. Health Educ Q. Spring 1984;11(1):1–47.doi:10.1177/109019818401100101 6392204

[19] Rosenstock IM. The Health Belief Model and Preventive Health Behavior. Health Education Monographs.1974;2(4):354–386. doi:10.1177/109019817400200405 299611

[20] Scarinci IC, Pandya VN, Kim YI, Factors Associated with Perceived Susceptibility to COVID-19 Among Urban and Rural Adults in Alabama. J Community Health. Oct 2021;46(5):932–941. doi:10.1007/s10900-021-00976-3 33751308 PMC7983968

[21] Dumont A. Changes in the U.S. Economy and Rural-Urban Employment Disparities.https://www.federalreserve.gov/econres/notes/feds-notes/changes-in-the-us-economy-and-rural-urban-employment-disparities-20240119.html

[22] Montenovo L, Jiang X, Lozano-Rojas F, Determinants of Disparities in Early COVID-19 Job Losses. Demography. Jun 1 2022;59(3):827–855. doi:10.1215/00703370-9961471 35583671 PMC9177772

[23] Cho SJ, Lee J, Winters JV. Rural areas and Middle America see smaller employment losses from COVID-19. 2020:

[24] Greteman BB, Garcia-Auguste CJ, Gryzlak BM, Rural and urban differences in perceptions, behaviors, and health care disruptions during the COVID-19 pandemic. J Rural Health. Sep 2022;38(4):932–944.doi:10.1111/jrh.12667 35466479 PMC9115219

[25] Anyamele OD, McFarland SM, Fiakofi K. The Disparities on Loss of Employment Income by US Households During the COVID-19 Pandemic. J Econ Race Policy. 2022;5(2):115–133. doi:10.1007/s41996-021-00086-1 35300312 PMC8280380

[26] Callaghan T, Lueck JA, Trujillo KL, Ferdinand AO. Rural and Urban Differences in COVID-19 Prevention Behaviors. J Rural Health. Mar 2021;37(2):287–295. doi:10.1111/jrh.12556 33619836 PMC8013340

[27] Probst JC, Crouch EL, Eberth JM. COVID-19 risk mitigation behaviors among rural and urban community-dwelling older adults in summer, 2020. J Rural Health. Jun 2021;37(3):473–478. doi:10.1111/jrh.12600 34096648 PMC8242629

[28] Lin G, Zhang T, Zhang Y, Wang Q. Statewide Stay-at-Home Directives on the Spread of COVID-19 in Metropolitan and Nonmetropolitan Counties in the United States. J Rural Health. Jan 2021;37(1):222–223. doi:10.1111/jrh.12464 32391620 PMC7272888

[29] Gross B, Opalka A. Too Many Schools Leave Learning to Chance During the Pandemic. 2020. https://crpe.org/too-many-schools-leave-learning-to-chance-during-the-pandemic/

