# Supplementary material for: Impact of COVID Pandemic on Financial Burdens in Metro and Non-Metro Regions: Findings from a Cross-Sectional Survey
Source: Res Sq. 2025 Jul 15:rs.3.rs-6874039. Preprint. [Version 1] doi: 10.21203/rs.3.rs-6874039/v1 (PMC12288524; doi:10.21203/rs.3.rs-6874039/v1)
Supplement: Supplement 1 [file NIHPPrs6874039v1-supplement-1.pdf]

Supplementary Files

This is a list of supplementary files associated with this preprint. Click to download.

- Supportinginformationv2.0.docx
- Supportinginformationv3.0.docx
